# Physicochemical and Biological Indicators of Soils in an Organic Farming System

**DOI:** 10.1155/2021/9970957

**Published:** 2021-09-02

**Authors:** Beybit Nasiyev, Tursunay Vassilina, Ainur Zhylkybay, Vladimir Shibaikin, Akmarzhan Salykova

**Affiliations:** ^1^Zhangir Khan West Kazakhstan Agrarian-Technical University, Uralsk, Kazakhstan; ^2^Kazakh National Agrarian Research University, Almaty, Kazakhstan; ^3^Saratov State Vavilov Agrarian University, Saratov, Russia

## Abstract

In developed countries, the ideas of ecological agricultural production, continuous cycle, and waste-free production technologies have gained popularity. The effect from the production and consumption of ecological agricultural products is determined by the least harm to the environment, increasing the competitiveness of products, and receiving additional profit from increasing prices for higher quality products. The production of organically safe products is based on the principle of biologization, i.e., the widespread use of biological preparations, a high proportion of legumes (sources of nitrogen), and avoiding chemical plant protection products, transgenic plants, and genetically modified organisms (GMOs). This study aims to increase the productivity of safflower and improve the physicochemical and biological indicators of dark chestnut soils through the use of biologized technologies in the organic farming system. Standard methods for assessment and statistical analysis of physical and chemical parameters of soils were carried out in zone 1 of West Kazakhstan. This made it possible to identify the most optimal technology for the cultivation of safflower. The study results showed that under the influence of the phytomeliorative action of safflower in the 0–20 cm layer of dark chestnut soils, one could note an increase in the content of nitrate nitrogen by 5.95%, an increase in the content of mobile phosphorus by 5.22%, and soil loosening by 0.010 g/cm^3^, with the structure of the soil being 64.43%. Strong biological activity of the soil was established by the crops of safflower. The highest yield of safflower oil about 0.23 t/ha with an oil content of 30.1% was obtained using the biologized technology option. The use of biological technology, along with biological yields, increases oil yield by 0.06 t/ha or 28.06%.

## 1. Introduction

Agricultural intensification based on the use of mineral fertilizers and plant protection chemicals gives rise to many problems. Some of the main problems are the increase of environmental pollution, the depletion of biodiversity, soil exhaustion, and soil degradation. These problems are exacerbated by global climate change. There is a sharp increase in the number of harmful agents migrating from other continents and zones. New pests, weeds, and diseases appear in the fields. Due to the scale of the use of chemical pesticides, these harmful agents become resistant to chemically active substances. Every year, it becomes clearer that many problems can be eliminated only with the help of microbiological preparations and bio-organic fertilizers [1–3].

In Kazakhstan, organic products account for 0.1% of all products consumed in the country. Thus, out of 62 million agricultural lands used in the Republic, 26 million hectares are unfavorable in terms of erosion and salinity. While in the 1980s, Kazakhstan had 35 million hectares of arable land, at present, 20 million hectares are used by agricultural formations and the rest is not suitable for agricultural use due to degradation. More than 15 million hectares without sowing activities turn into reservoirs of pathogens and pests. All these problems can be largely solved with the effective use of biological products. Therefore, the main task in planning and using intensification factors should be to preserve the environment and increase soil fertility, which is a necessary basis for the implementation of advanced agricultural technologies and obtaining stable, environmental-friendly yields [4–6].

The biologization of agriculture, i.e., the process aimed at the predominant use of biological factors rather than chemical and technical factors to increase the economic efficiency of agricultural production, is becoming the main direction for increasing soil fertility and obtaining high yields of crops. According to economists, there is an annual turnover of 85–90 billion dollars in organic farming. Biological preparations steadily increase yields by 20–25%, while significantly reducing plant disease [1]. Organic practices in agriculture are currently used in 160 countries of the world. Organic agriculture laws work in 84 countries; in dozens of countries, such bills are drafted. Economists estimate that based on current gross turnover in organic agriculture, which amounts to $85 to $90 billion per year, this amount is projected to reach $200–250 billion by 2020 [7]. On November 27, 2015, the Law of the Republic of Kazakhstan “On production of organic products” was adopted. The Act established the legal, economic, social, and organizational base for the management of organic agriculture. This legislation is aimed at the rational use of the soil, promotion of healthy diets, and protection of the environment [8]. Currently, Kazakhstan is in the process of adapting the international standards of Codex Alimentarius, as well as those of IFOAM, and is also gaining the international experience as an organic agricultural producer at the local level.

Due to the recent climate changes, the creation of new safflower varieties, adaptable, resistant to unfavorable environments, and highly productive, require the development of technology for the use of biological products. They mostly contribute to increasing the productivity of culture. In agronomic science, there are studies on the effect of biological preparations on oilseeds.

There are studies of biological preparations on crops in agronomic science. Babenko with co-authors used signaling molecules of quorum-sensing bacterial cells to stimulate the growth of wheat. Autoinducer *N*-acyl homoserine lactone activated rhizosphere microflora, which positively affected wheat biomass and grain yield as a result [9]. Meta-analysis of the efficiency of microbial fertilizers based on a complex of microorganisms showed that the best inoculum for plants is a combination of arbuscular mycorrhiza, nitrogen fixators, and phosphate mobilizers. The effectiveness of such a complex was shown in 92% of 112 field experiments. Thus, the contribution to the yield is significant, and its variability is low [10]. The use of rizoagrin ensured an increase in wheat yield to 0.41 t/ha (13.5%) in comparison with the untreated control, while application of flavobakterin in combination with humates resulted in an increase of 17.1% [11]. Application of ekstrasol to seeds of spring wheat followed by vegetation under the conditions of nitrogen-free background only promoted an increase in spring wheat productivity to 26% on average. It has been demonstrated that the complex use of nitrogen fertilizer in a dose of 45 kg/ha and biological products of endophytic bacteria made it possible to increase the grain efficiency of spring-sown wheat by 1.6–2.1 times [12].

There are studies on the application of organo-biological fertilizers in a system of biologized technology and on safflower crops. In experiments carried out at CollegeFarm, Rajendranagar, Hyderabad (India), organo-biological fertilizers gave high yields of safflower seeds. Nutrient uptake, gross yield, and net income were recorded with S7 (soil test-based fertilizers + vermicompost @ 2 t ha^−1^) and they were significantly superior to S6 (RDF + vermicompost @ 2 t ha^−1^) followed by S5 (soil test-based fertilizers + FYM @ 5 t ha^−1^), S4 (RDF + FYM @ 5 t ha^−1^), S3 (soil test-based fertilizers), and S2 (RDF) [13].

In experiments carried out at the Sarayonyu experimental area of Selcuk University (Dincer, Turkey), the application of organic fertilizers increased the oil content of safflower kernels compared to the application of diammonium phosphate and without it [14]. According to the scientists, a strategy to mitigate the detrimental effects of salinity on safflower by applying antioxidants and enzymes such as glycine betaine (GB) is promising [15].

According to Tolmachev's recommendation in the Volgograd region of Russia for cultivation with biological crop rotations, it is advisable to sow safflower in earlier terms with the use of biopreparations and growth regulators [16]. In the Ivanov and Tolmachev research, when sowing seeds treated with organic preparations of Biodux (1 ml/t) at optimal early sowing periods (the temperature in the layer 0–10 cm: 6 + 80°C), the yield capacity of safflower varieties Kamyshinsky 73 was within 0.6–1.0 t/ha, with oil content 27–30% [17]. The profitability level, in this case, reached 155.2%, and the energy efficiency coefficient was 2.85. In Kazakhstan, the bio-organic preparation Avibif (1 l/ha) showed high efficiency on safflower crops [18]. Ivanchenko and Belikina's research under conditions of the Federal Research Center for Agro-Ecology of the Russian Academy of Sciences revealed the efficiency of safflower seed disinfection with a mixture of preparations—biofungicide Vincit (1.5 l/t) + biostimulator Fertigrain Start (0.5 l/t) [19]. This variant was the most profitable (16.5%) with a yield of 1.2 t/ha. In fertilizer application, 4 and 7 ton h^−1^ vermicompost showed a higher seed yield of safflower (*Carthamus tinctorius* L.) [20].

Safflower (*Carthamus tinctorius* L.) belongs to the Asteraceae family [21]. It is originally from Egypt and India. It is rich in vitamin E [22, 23]. Safflower is considered an important agricultural crop. Safflower seeds are an important alternative to oil crops because of their high oil content (27–32%) and rich linoleic acid content (55–70%). Cold-pressed safflower oil has high nutritional and pharmaceutical values due to the significant amount of biologically active compounds and essential fatty acids. The oil is known as a valuable source of alpha-tocopherol, which shows the highest vitamin E activity and therefore has many health benefits such as prevention and treatment of hyperlipemia, arteriosclerosis, and coronary heart disease [24]. Positive effects of the safflower seed extract (SSE) on the human cardiovascular system have been proven in the study by Singh and Nimbkar [25].

Antinutritional factors (ANF) present in safflower seeds, responsible for the characteristic tart or bitter taste, affect the livelihood of animals with a single-chambered stomach. Appropriate processing methods help to reduce the undesirable effects of ANF and thereby improve the feed and nutritional value of safflower. ANF in safflower seeds and their well-organized extraction methods can add a positive approach in medical science for their further application in the pharmaceutical industry [26].

Safflower seeds and their products play an important role in the food complex of the country. Cultivation of safflower is also relevant in the climatic conditions of West Kazakhstan, characterized by high heat supply and a long growing season. Earlier, safflower used to be sown more in East Kazakhstan and Almaty regions, now it is increasingly produced in the north, western regions, and south. There is a very strong demand for safflower, it is taken up by all neighboring countries, and it is exported very well to China [4, 5].

Safflower is one of the most drought-tolerant crops throughout the world. Its deep and pivoting root system allows it to explore deeper soil layers, enhancing its ability to extract water and nutrients that are not available for most crops [27].

In our opinion, in this region, safflower can occupy a certain niche in the formation of biologized agricultural landscapes. The role of safflower in increasing the production of vegetable protein and oilseeds is significant. Its cultivation allows a more rational use of the potential of lands in arid regions with a decrease in the cost of oilseed production [28].

The main aspect in a new field in agriculture called organic farming, a way not only to obtain environmental friendly products but also to protect, restore, and enrich natural biological diversity which may gradually be lost due to the extreme enthusiasm of humanity in using chemical products to achieve high production rates, is the application of agricultural landscapes of crops used as phytomeliorants. Along with high drought tolerance and yield, the phytomeliorative role of safflower is of great importance. In the works of many scientists, there is scientific evidence of the positive role of safflower as a green fertilizer in improving soil fertility of safflower [29]. Postnikov suggests the cultivation of safflower as a phytomeliorant in contaminated soils with its sequential removal [30] to clean soils from heavy metals. The results of tests on safflower, when used as a phytomeliorant, proved convincingly high efficiency of the accumulating ability of plants to reduce the content of heavy metals in the root layer of soil. Based on these studies, Sarto et al. noted the resistance of safflower to soil compaction and highlighted safflower as a species capable of reducing volumetric soil compaction [31]. In this study, the Q1/2 index was higher than 1.77 and 1.55 for the IMA-2106 and IMA-4904 genotypes, respectively.

The soils of West Kazakhstan, depending on their genesis, confinement to different types of landscapes, the prevailing moisture conditions, and redox conditions, differ significantly in the intensity of microbiological processes, which ultimately determines their ecological state. The use of organic and microbiological preparations is promising in the increase of microbiological activity of soils in West Kazakhstan. Soils contain natural reserves of plant nutrients, but these reserves are largely in forms unavailable to the plants, and only a minor portion is released each year through biological activity or chemical processes. This release is too slow to compensate for the removal of nutrients by agricultural production and to meet crop requirements. Therefore, fertilizers are designed to supplement the nutrients already present in the soil. The use of chemical fertilizers, organic fertilizers, or biofertilizers has its advantages and disadvantages in the context of nutrient supply, crop growth, and environmental quality. The advantages need to be integrated to make optimum use of each type of fertilizer and achieve balanced nutrient management for crop growth. Biofertilizers are the alternative sources needed to meet the nutrient requirement of crops. In biofertilizers, beneficial bacteria such as Azotobacter, Azospirillium, Rhizobium, and Mycorrhizae are very essential in crop production. Biofertilizers can also make a plant resistant to unfavorable environmental stresses [32, 33].

In a study by Kireeva et al., when applied to oil-contaminated soils, biological preparations ensured an increase in the yield of spring wheat by increasing the microbiological activity of the soil (Table 1) [34].

In Ivanov et al.'s research in Zavolzhye in Volgograd, the use of biological preparations helped in increasing the yield of safflower to 1.04 t/ha of oilseeds with an oil content of 29.0% [17].

According to Malusà et al., biofertilizers can play a key role in developing an integrated nutrient management system, sustaining agricultural productivity with low environmental impact [35]. However, there are no data on the study of biological technologies of safflower cultivation on the biological activity of dark chestnut soils. There is no scientific information about the effects of the biological products on the quantitative and qualitative composition of the beneficial microflora of dark chestnut soils. The objective of the research is to identify changes in the physicochemical and biological indicators of the soil cover, productivity, and quality of safflower under the influence of different technologies for the formation of agricultural landscapes and for rational management of agrocenoses.

## 2. Materials and Methods

### 2.1. Research Design

This research was conducted in 2020 at the Zhangir Khan West Kazakhstan Agrarian and Technical University (Kazakhstan) with the initiative of the Science Committee of the Ministry of Education and Science on the territory of the farm “Daukara” of the West Kazakhstan region.

**Figure 1:**
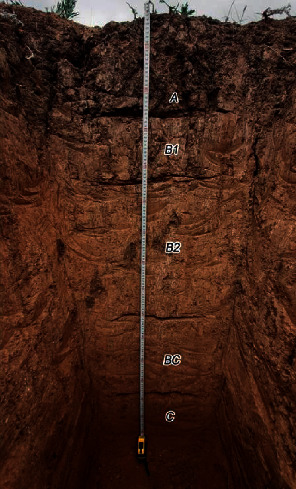
A section of dark chestnut soils.

According to morphological characteristics of genetic horizons of the profile and agrochemical indicators of the arable layer of soils, the experimental plots typical for zone 1 of West Kazakhstan show dark chestnut medium-loamy soils (*Haplic Kastanozems*) (Figure 1).

**Figure 2:**
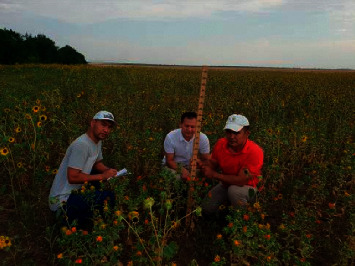
A scheme of the field experiment.

The object of the study was agrolandscapes of safflower (*Carthamus tinctorius* L.) (Figure 2).

The scheme of the field experiment is presented in Table 2.

In this study, two technologies for the formation of safflower crops were comparatively studied:

1. First is the traditional technology (control variant) without the use of biological agents. In this technology, nitrogen and phosphorus fertilizers are used in minimal doses of N_20_P_20_ before sowing safflower. The following mineral fertilizers were used: ammonium nitrate NH_4_NO_3_ and double superphosphate Ca(H_2_PO_4_)_2_.
2. Second is the biologized technology using biological organic products available in the market for farmers: biostimulator Biodux, biofungicide Orgamica S, and bio-organic fertilizers Organit N and Organit P. Biological products were applied in two doses: for dressing safflower seed material at a dose of 10 l/t and for the treatment of safflower plants in the 3-4 leaves phase by spraying on the crops. The consumption of the working solution is 300 l/ha.

In both variants of the experiment, the adopted tillage system in zone 1 of Western Kazakhstan was applied.

The zoned Akhram safflower variety was used in the experiments. The seeding rate was 500 thousand viable seeds per hectare.

Safflower was harvested using the solid method in the phase of complete ripeness of safflower, bringing the crop to 10% moisture content at 100% purity. Harvesting was carried out using pin-drum harvesters at a reduced speed of 750–800 rpm.

### 2.2. Stages of the Study

The area of divisions is 50 m^2^, the repetition was threefold, and the location of divisions was random.

#### 2.2.1. Soil Sampling

To determine the impact of technology on the indicators, soil samples were collected from the territories of zone 1 of West Kazakhstan with dark chestnut soils (Haplic Kastanozems) in the 0–10 cm and 10–20 cm layers. To identify changes in soil parameters, by comparison, soil samples were collected from control plots (traditional technology) of the 0–10 cm and 10–20 cm layers. The selection was repeated 4 times.

Under laboratory conditions, the content of nitrate nitrogen and mobile phosphorus in the soil was determined by analyzing soil samples.

In the experiments, research was carried out to study the effects of biologized technology on the biological activity of dark chestnut soils.

#### 2.2.2. Physical and Chemical Indicator Analyses

The identification of changes in the physical and chemical indicators of agricultural landscapes was carried out both in the field and by organizing laboratory analyses using accepted modern methods.

*(1)The Biometric Method*. The organization of observations of the onset of phenological phases accounting for the growth and development (height, density of crops, and structure of the yield) of safflower was carried out according to generally accepted methods [36]. The phenology study allowed us to determine the main phases of crop development, especially the time of full flowering under different technologies, the duration of flowering, and physiological ripening, as the success of harvesting depended on it. The main growth and development phases of safflower were established in the research: sprouting, 3-4 leaves, stemming, basket formation, flowering, and ripening phases.

The study of the growth dynamics (height) allowed us to determine the period of the most intensive growth. The height of safflower plants was determined in 10 locations on a plot in 2 noncontiguous replications of the experiment. Finding the density of plants on sprouts and before harvesting allowed establishment of the influence of the factor under study on the condition of sprouts and plant fallout during the growing season. The sprouting density and the number of plants that survived harvesting were determined by counting sprouts and safflower plants before harvesting on 4 permanent plots of 0.25 m^2^ in 2 noncontiguous replications of the experiment.

The yield structure of safflower was determined by breaking a 1 kg sheaf into its parts—stem, leaves, and anthodium. To determine the biological yield, the number of productive anthodium, anthodium diameter, number of seeds per anthodium, and weight of 1000 safflower seeds were calculated.

*(2)The Extraction Method for the Determination of Crude Fat*. Fat content in the seeds was calculated to determine the oil content of safflower. The oil content of seeds means their crude fat content and accompanying fat-like substances transferred together with fat into the ether extract from the examined seeds. The oil content in safflower seeds was determined by the extraction method by extracting crude fat from seeds with an appropriate solvent in a Soxhlet apparatus. To determine crude fat content in safflower seeds, about 40 g of seeds were isolated, weighed to the nearest 0.01 g, and sieved through two sieves with hole diameters of 3 and 0.5 mm in the upper and lower sieves, respectively. The seeds, freed from the abovementioned impurities, were transferred into a porcelain cup and dried at 100–105°C for 1 h. Subsequently, extraction was carried out. The duration of sunflower seed extraction was 22–24 h. The end of extraction was determined by the absence of fat in the extraction-completed sample. At the end of extraction, the ether was stripped and the oil was dried in a desiccator at 100–105°C until weight constancy [37].

*(3)The Determination of Nitrate Nitrogen Content in Air-Dry Soils Using Calcium Chloride as an Extractant*. Determinable soil nitrogen fractions were obtained by extraction of air-dry soil samples with calcium chloride (СаС1_2_) solution of 0.01 mol/dm^3^. Inorganic nitrogen compounds (nitrate (+nitrite) and ammonium) were determined directly in the soil extract, using automated spectrometric methods of segmented flow analysis. All operations of preparation of the extract for measurement, graduation of the spectrophotometer, and measurement of the concentration of the determined fractions were carried out in a closed flow-through inside elastic silicone tubes. The flow of extract and reagent solutions was carried out with a pump forcing the solutions by rhythmic pressure of the silicone tubes. Mixing of the extractant with the reagents took place in the reaction coils with air bubbles. The colored solution entered the flow cell of the spectrophotometer, and the result of the light absorption was recorded with a chart recorder. The amount of nitrogen in the soil organic matter soluble in extracting solution with (СаС1_2_)-0.01 mol/dm^3^ was determined by the calculation method [37].

*(4)The Photometric Method for Determination of Mobile Phosphorus*. The method is based on the extraction of mobile phosphorus and potassium compounds from the soil with a solution of ammonium carbonate at a concentration of 10 g/dm^3^ at a soil: solution ratio of 1 : 20 and subsequent determination of phosphorus in the form of a blue phosphorus-molybdenum complex on a photoelectric colorimeter and determination of potassium on a flame photometer [37].

*(5)The Cylinder Method*. Soil density was determined in the field using N.A. Kachinsky drill cylinders. In the field, samples were taken from the soil horizon with a drill cylinder with a volume of about 500 cm^3^. Simultaneously, to determine the moisture content, soil samples were collected in weighing bottles. In the laboratory period, the soil was dried at 105°C to constant weight. Knowing the weight of the weighing bottle with dried soil and the weight of the empty bottle, we found the weight of air-dry soil. Then, by dividing the mass of dry soil by its volume (the volume of the ring), the density of the soil was established.

*(6)Dry Soil Sieving Method*. The assessment of the structural state of the soil was carried out by aggregate analyses using the dry sieving method. Based on the results of the aggregate analysis, the structural coefficient (С_str_) was calculated, which is understood as the ratio of the number of aggregates from 0.25 to 10 mm (in %) to the total content of aggregates less than 0.25 and more than 10 mm (in %). The bigger the С_str_, the better the soil structure. To assess the structural state of soils, we used a scale developed by Dolgov and Bakhtin [37].

*(7)The Method of Flax Linen Decomposition (Application Method).* The method is based on the determination of the biological activity of the soil by the intensity of cellulose decomposition [38]. The intensity of cellulose decomposition was assessed by the loss in weight of linen cloth. To assess the biological activity of soils on the decomposition of cellulose in the autumn period (% of decomposed linen in 2 months), we used the Zvyagintsev scale: very weak (<10), weak (10–30), medium (30–50), strong (50–80), and very strong (>80).

*(8)Statistical Analyses*. Statistical processing of the study results was carried out by the method of dispersion analysis [39], using the Statistica 6.0 software.

## 3. Results and Discussion

### 3.1. Features of Growth and Development of Safflower in Biologized Cultivation Technology

One of the most important structural elements that determine the productivity of safflower is the plant density in crops, determined for different climatic zones for their cultivation. Science and practice show the optimal number of plants per unit area of the field provides for the best use of environmental factors by crops. Water, light, temperature, and soil fertility are the most important factors that determine the formation of plant density in crops. These factors have a significant impact on seed germination, the emergence of seedlings, and field germination, which is an important production indicator [40].

In the phase of full germination of safflower and before harvesting, we determined, respectively, the field germination of seeds and considered the plant density. Since sparse crops cannot guarantee a good harvest, high field germination is the most important indicator for a good yield. As our research has shown, safflower is characterized by fairly high field germination of seeds. In studies, the field germination of safflower, depending on the cultivation technology, equaled 91.5–92.8%. The highest completeness of seedlings is provided when the seeds are treated with biological preparations.

### 3.2. Results of Phenological Observations

In 2020, the development of safflower plants depended on cultivation technology. When sowed on April 27^th^ in the two studied cultivation technologies, safflower seedlings appeared on May 10, i.e., 13 days after sowing. Starting from the budding phase, there was a difference in the rate of development of safflower depending on the variants of the experiment. With traditional technology, the budding phase began on July 1, or 51 days after germination. When using biologized technology, i.e., with the combined use of the biological product Biodux, biofungicide Orgamica S, and bio-organic fertilizers Organit N and Organit P (biologized technology) by seed treatment and treatment during the growing season of safflower (foliar feeding of plants in the 3–6 leaves phase), we noted an early onset of the budding phase by 2 days compared to the safflower plants of the control variant.

In this variant, the budding phase began on June 28, or 49 days after the emergence of full shoots. This tendency of the development of safflower according to the two variants continued in the flowering phase. According to the variants of the experiment, safflower entered the flowering phases on July 16th and18th. For the variant of the biologized technology, the flowering phase began 2 days earlier than that of the control variant. The flowering was even and lasted 34 days in the version of biologized technology. When using traditional technology, the duration of the flowering period and ripening lasted 37 days, that is, 3 days longer than in the case of using biological products. Full ripening in 2020 in the control variant was reached on August 25, while the total duration of the growing season was 107 days. The use of biological products (biological product Biodux, biofungicide Orgamica S, and bio-organic fertilizers Organit N and Organit P (biologized technology)) reduced the duration of the safflower vegetation period in zone 1 of West Kazakhstan compared to traditional technology by 3 days. With the studied biologized technology, the duration of the growing season of safflower was 104 days. We should note the importance of reducing the duration of the growing season and the even character of safflower ripening for the timely and high-quality organization of harvesting.

### 3.3. Safflower Plant Growth Dynamics

One of the indicators characterizing the state of agrocenosis is the height of the plants. Observations of the dynamics of the linear growth of safflower showed that the height of the plants depended on the weather conditions of the growing season and the cultivation technology. The analysis showed that at the beginning of the growing season, safflower has a low growth rate in height. The most intense growth of plants in height was observed in the period from stemming to the beginning of flowering. Then, the growth rates decreased, and by the phase of the onset of ripeness, the plants in the studied variants had the highest height.

In studies from the stemming phase, the difference in the growth of safflower plants depending on the cultivation technology was noted. In the stemming phase, the height of the plants in the control variant was 20 cm, and when using the biologized technology, the plants had a height of 24 cm. Thus, the difference between the plant heights of the studied variants was 4 cm.

**Figure 3:**
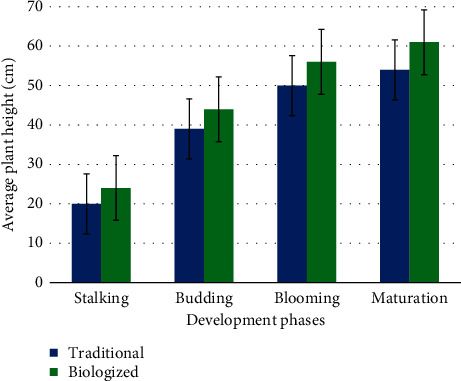
Dynamics of safflower growth depending on cultivation technology (cm).

In the budding phase, the height of safflower plants, according to the experimental variants, was within 39–44 cm, and by the flowering phase, safflower plants had a height of 50–56 cm. The combined use of the biological product Biodux, biofungicide Orgamica S, and bio-organic fertilizers Organit N and Organit P (biologized technology) ensured the maximum growth of safflower plants in comparison with the control variant (traditional technology). By the ripening period, the safflower plants in the biologized version of the technology had reached 61 cm. Before harvesting, the plant height of the control variant was 54 cm; that is, the plants of this variant were 7 cm shorter than the plants of the biologized technology variant (Figure 3).

### 3.4. Weed Infestation of Crops

Weeds cause great damage to safflower crops. Safflower, when sown early, due to the relatively fast growth rate of the stem can resist weeds [41]. The weed infestation of rainfed lands in West Kazakhstan is one of the major obstacles to further increase safflower yields.

As shown by the accounting data in our studies in 2020, the highest weed infestation of safflower crops was noted in the control variant in which the traditional technology was used. Thus, in the 3–6 true leaves phase, in the traditional (control) variant, the number of weeds per m^2^ was eight with a wet weight of 29.45 g/m^2^. In the biologized technology variant, the weed infestation of crops constituted six weeds with a wet weight of 23.35 g/m^2^. The weeds found in the experimental plots were the following: *Capsella*, *Chenopodium album*, *Fallopia convolvulus*, *Amaranthus retroflexus, Raphanus raphanistrum, Echinochloa crus-galli, Convolvulus arvensis*, and *Cirsium arvense.*

In the flowering phase, the greatest weed infestation of safflower crops was found in the control variant using traditional technology. Here, 37 weeds with a wet weight of 179.35 g/m^2^ were found per m^2^. In the case of using biological preparations, the number of weeds was 22 with a wet weight of 110.77 g/m^2^. In 2020, rains in the period of flowering and grain filling of safflower contributed to the growth and development of weeds. During the harvesting period, compared with the flowering phase, the number of weeds in the control variant increased by 9 and the weed infestation in that variant was at the level of 46 weeds/m^2^. The wet weight of the weeds was 230.75 g/m^2^. With the combined use of the biological product Biodux, biofungicide Orgamica S, and bio-organic fertilizers Organit N and Organit P, only 33 weeds per m^2^ with a wet weight of 170.25 g/m^2^ were found during safflower ripening.

### 3.5. Influence of Cultivation Technology on the Formation of Elements of Productivity and Oil Content of Safflower

When grown in various soil and climatic conditions, crops show noticeable features of the formation of elements of crop productivity [42]. In safflower, among these indicators, it is necessary to single out important conditions for crops such as the density of standing plants preserved for harvesting (pcs/m^2^), the number of flower heads per plant (pcs), the number of completed seeds per plant (pcs), the number of completed seeds in 1 flower head (pcs), the weight of seeds from one inflorescence (g), and the weight of seeds from one plant (g).

Elements of technology significantly affect the yield of any crop. Incorrectly selected technology parameters can lead to the formation of low productivity indicators of safflower crops, which in turn can affect the yield of oilseeds. In the study, the best indicators of the elements of the structure of the crop and the yield of safflower were established using biologized cultivation technology.

Cultivation technologies have had a significant impact on the safety of safflower plants by the end of the growing season. In studies with the combined use of the biological product Biodux, biofungicide Orgamica S, and bio-organic fertilizers Organit N and Organit P (biologized technology) by seed dressing and processing during the growing season of safflower (foliar feeding of plants in the 3–6 leaves phase), the good capacity for survival until the harvest was noted in safflower crops compared with the control variant (traditional cultivation technology). By the time of harvesting of the variant using biologized technology, out of 46.40 pieces/m^2^ of emerged plants, 88.04%, or 40.85 pieces/m^2^, were preserved, while out of 45.75 pieces/m^2^, 38.45 pieces/m^2^, or 84.04%, were preserved in the control variant. Before harvesting in the sowing field with the use of biologized technology, 24 pieces/m^2^ of plants were more preserved than in the control variant. The combined use of the biological product Biodux, biofungicide Orgamica S, and bio-organic fertilizers Organit N and Organit P (biologized technology) by seed dressing and treatment during the growing season provided the number of productive flower heads in safflower plants to be up to 17.0 pcs per plant in comparison with the control variant. With an average diameter of flower heads (2.18–2.41 cm) when using biologized technology, compared with the control variant, the number of seeds per flower head was over 1.1. The use of biological preparations also contributed to an increase in the weight of 1000 seeds from 42.70 to 43.15 g (Table 3).

Studies have shown that the oil content of safflower seeds varies under the influence of environmental conditions prevailing during the growing season and elements of cultivation technology, which confirms the conclusions of other scientists [43]. In the study, the fat content of seeds was reduced with the use of traditional technology by 28.7%. In 2020, as a result of comparative studies on safflower oil content, an increase in oil content up to 30.1% was revealed when using biologized technology. In this study, the highest oil collection of 0.23 t/ha was obtained with the combined use of the biological product Biodux, the biofungicide Orgamica S, and the bio-organic fertilizers Organit N and Organit P (biologized technology) by seed treatment and plant treatment during the growing season. The use of traditional techniques along with biological yield reduces the oil yield by 0.06 t/ha, or 28.06% (Table 4).

The results of statistical analysis by the T-criterion for two independent samples showed a high probability of dependence of yield, oil collection, and oil content on the cultivation technology. Levene's test is not statistically significant (*p*=0.0561 − 1). Student's *t*-test confirms the differences in the average indicators by type of technology (*p* level) less than 0.05. Consequently, the differences in the mean values for the two variants for all indicators (yield, oil collection, and oil content) are statistically significant. The confidence level is at a high level of significance (*p*=0.004).

**Figure 4:**
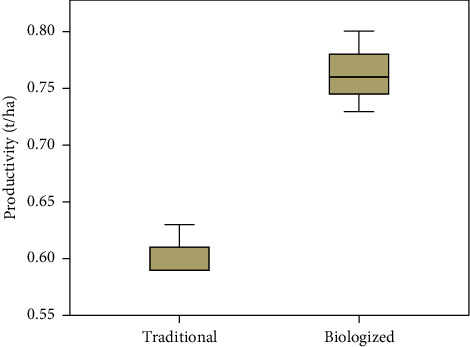
Safflower yield depending on cultivation technology in zone 1 of West Kazakhstan (t/ha).

Studies have established the highest yield of 0.76 t/ha with the combined use of the biological product Biodux, biofungicide Orgamica S, and bio-organic fertilizers Organit N and Organit P (biologized technology) by seed treatment and processing during the growing season. The use of traditional technology reduces the biological yield of safflower to 0.16 t/ha, or 21.05% (Figure 4).

Figure 4 shows that the values of the yield distribution in the box plot differ from each other. The maximum and minimum values for technologies do not overlap. There is a yield response depending on the cultivation technology. The yield distribution according to the traditional technology is asymmetric, and the median is shifted closer to the lower border of the box plot. Thus, the median yield value of traditional technology is biased towards the minimum value and can be a representative value, given the sample size.

The hull content of seeds is a quality indicator that needs to be reduced [43]. In this study, the hull content increased with the traditional cultivation technology up to 33.6%. The smallest indicator of hull content was noted when using the biologized technology (32.3%).

### 3.6. Study of the Phytomeliorative Role of Safflower in Dark Chestnut Soils of West Kazakhstan in the System of Organic Farming

Modern agriculture in Kazakhstan is currently in a situation where it is necessary to solve the problems of restoring soil fertility associated with a sharp reduction in fields occupied by fodder grasses and green manure crops, a decrease in the use of organic fertilizers, and a violation of crop rotation systems [44]. Therefore, to maintain or restore agricultural soils with the factor of fertility, it is necessary first of all to develop agricultural practices using new and traditional green manure crops. At present, the agroecological role of green manure crops in maintaining the balance of the main nutrients in the topsoil is difficult to overestimate. At the present stage of the development of agriculture, it is necessary to search for new crops with phytomeliorative properties.

Sideration has been studied by many scientists, and its main research was carried out with crucifers and legumes. As a rule, researchers note a generally positive trend in the increase in the content of nitrogen, available phosphorus, and potassium in the arable layer [29, 33, 45, 46].

In our study carried out to clarify the phytomeliorative role, data were obtained on the microbiological activity of the soil after plowing safflower.

To assess the phytomeliorative role of safflower in improving the fertility of dark chestnut soils in zone 1 of West Kazakhstan, this crop was sown on the fields of the Daukara peasant farm on April 27, 2020.

**Figure 5:**
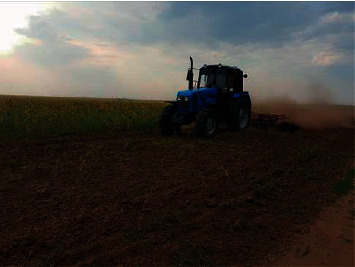
Sowing safflower silage into the soil.

By the flowering period, the height of safflower plants during cultivation for plowing as a phytomeliorant in the stemming phase had reached 22 cm, and in the budding phase, it equaled 45 cm. Safflower bloom began on July 16. During that period, the green mass of safflower was plowed into the soil with disc harrows to a depth of 18–20 cm (Figure 5).

Before plowing, the yield of the green manure mass of safflower was determined. In this study, safflower plants in zone 1 of West Kazakhstan in the flowering phase formed a green mass of 117.7 c/ha. By the flowering phase during the plowing period, the safflower plant had reached a height of 60 cm. During the plowing period of green mass, the content of nitrogen and phosphorus in plants was determined. As shown by the agrochemical analysis data, by the time of plowing, the composition of the green mass of safflower included 1.72% nitrogen and 3.30% phosphorus in dry weight.

Most soils naturally contain insufficient amounts of nitrogen, phosphorus, and potassium available to plants, as well as other nutrients. Besides, every year, a significant amount of these elements is alienated from the soil with harvesting. Moreover, most of them are still lost due to leaching and volatilization or are fixed in the soil, passing into forms inaccessible to plants. The reserves of these elements can be replenished only artificially, through the use of fertilizers, including green manure.

By the fall of 2020, soil samples had been taken and analyzed to assess the phytomeliorative effect of safflower on dark chestnut soils.

As shown by the data of agrochemical analysis, safflower contributed to an increase in the content of nutrient mineral elements in the soil. Thus, by autumn, on a plot plowed with safflower, an increase in the content of nitrate nitrogen and mobile phosphorus was noted in comparison with the content of these elements in the spring period before sowing.

In the 0–20 cm layer of dark chestnut soils, under the influence of the phytomeliorative action of safflower, an increase in the content of nitrate nitrogen from 5.04 to 5.34 mg/100 g of soil, or by 5.95%, was noted by autumn.

A similar trend is observed in the content of mobile phosphorus. During the spring-autumn period, in the 0–20 cm layer of dark chestnut soils, the content of mobile phosphorus had increased from 1.15 to 1.21 mg/100 g of soil, or by 5.22% (Table 5).

Under the influence of cultivated crops on arable land, soil density, its water-air properties, temperature, and nutritional regimes, microrelief, dynamics of vegetation cover, and quality of micropopulation and macropopulation of the soil can change.

The sowing of phytomeliorants has a positive effect on the agrophysical parameters of soils. This study confirms the findings of other authors [29, 31, 45]. If in the root-inhabited layer of 0–20 cm in the spring, the soil density was at the level of 1.30 g/cm^3^, then by autumn, there was a tendency for the density of the soil to decrease in layers of 0–10 and 10–20 cm. During the growing season, in the 0–20 cm layer, soil loosening by 0.010 g/cm^3^ was noted.

Analysis of the dynamics of the structural and aggregate composition of dark chestnut soils indicates some improvement in the structure of soils under the influence of the phytomeliorative action of safflower and a pronounced tendency to show recovery, noted during the observation period.

Due to the phytomeliorative effect of safflower, the dark chestnut soils of the experimental plots have good indicators in terms of the content of agronomically valuable aggregates and the structural coefficient. Thus, on dark chestnut soils in the autumn after sowing safflower, the soil structure in the 0–20 cm layer was 64.43% with a structure coefficient of 1.68. According to the accepted criteria, the soil has a good structure and good structural properties (Table 6).

Table 7 shows the results of this study in determining the microbiological activity of dark chestnut soils. As the research data show, on the variant with safflower, a very high rate of decomposition of linen is noted, and 2 months after laying, the total mass of the linen compared to the control variant was reduced by 55.75%. This corresponds to the biological assessment of soil activity as “strong.”

To establish the influence of forage crops on the biological activity of the soil, the intensity of cellulose decomposition was also determined in experimental plots under barley crops. As shown by the research data, barley, in comparison with safflower, has the least effect on the biological activity of dark chestnut soils. In experiments, by an average of four replicates, the level of decomposition of linen was 8.84% compared to the control variant. This indicates a very weak biological activity of this culture.

Thus, in field crop rotations under the conditions of zone 1 of the West Kazakhstan region, along with the use of a fallow field, it is advisable to introduce safflower as a green manure crop, which after plowing has a positive effect on the agrophysical, agrochemical, and biological indicators of dark chestnut soils.

## 4. Conclusion

1. Deterioration of physical, chemical, and biological indicators and degradation and desertification processes are the most common and significant complications in the management of agricultural landscapes in West Kazakhstan. They require correct decision-making due to their impact on the environment reducing the rate of production of safe crop products.
2. Physicochemical and biological indicators affect the quality of the soil, thereby affecting the ecological situation in agrocenoses and the potential productivity and state of agricultural landscapes.
3. To solve the problems of high quality and rational use, it is necessary to use biologized technologies for the formation of agrocenoses along with bio-organic preparations and fertilizers.
4. The basic principle of organic farming is the use of biologized technologies that improve the physical, chemical, and biological parameters of soils. This hypothesis is confirmed by the data of standard methods used for assessing the physicochemical and biological indicators of dark chestnut soils under safflower agricultural landscapes in the territories of zone 1 of West Kazakhstan.
5. These ideas and research data serve as a prerequisite for the development of comprehensive measures for the rational use of agricultural landscapes outside Kazakhstan, in countries and regions with similar agrocenosis management systems.

## Figures and Tables

**Table 1:** Yield of spring wheat on oil-contaminated soils (5% wt.) (c/ha).

Period after contamination, in months	Control	Biopreparations		
		Belvitamil	Phytosporin	Lenoil
—	17.8	23.0	24.0	20
2	1.2	4.5	5.0	—
12	2.0	15.6	16.0	13.0

**Table 2:** A scheme of the field experiment.

Variants	Traditional technology	Biologized technology^*∗*^
The essence of the technology	Only mineral fertilizers are used: NH_4_NO_3_ (ammonium nitrate) and Ca(H_2_PO_4_)_2_ (double superphosphate) at a dose of N_20_P_20_ before sowing	The biostimulator Biodux, the biofungicide Orgamica S, and bio-organic fertilizers Organit N and Organit P are used

^*∗*^The products are used for seed disinfection and spraying of safflower during the growing season in the phase of 3-4 true leaves.

**Table 3:** Structure of the elements of safflower yield depending on the cultivation technology in zone 1 of West Kazakhstan.

Technologies	Number of plants per m^2^, pcs	Number of productive flower heads per plant, pcs	Flower head diameter per plant, cm	Number of seeds per plant, pcs	Weight of 1000 seeds, g	Yield, ton/ha
Traditional (control) variant	38.45	15.00	2.18	24.40	42.70	0.60
Biologized variant	40.85	17.00	2.41	25.50	43.15	0.76

**Table 4:** Indicators of yield and quality of safflower depending on the cultivation technology in zone 1 of West Kazakhstan.

Technologies	Yield (ton/ha)			Oil harvest (ton/ha)			Fat content (%)		
	I	II	III	I	II	III	I	II	III
Traditional (control) variant	0.63	0.59	0.59	0.18	0.17	0.17	28.5	28.8	28.8
Biologized variant	0.76	0.80	0.73	0.23	0.24	0.22	30.2	30.0	30.1
*F* test		^*∗∗∗*^			^*∗∗∗*^			^*∗∗∗*^	

*F* test of significance: ^*∗∗∗*^*p* level <0.01. I, II, and III: experiment replicates.

**Table 5:** Phytomeliorative effect of safflower on the content of chemical indicators of dark chestnut soils.

Soil layer, cm	Nitrate nitrogen, mg/100 g of soil			Mobile phosphorus, mg/100 g of soil		
	Spring	Autumn	Difference	Spring	Autumn	Difference
0–10	4.88	5.10	+0.22	1.19	1.26	+0.07
10–20	5.20	5.57	+0.37	1.11	1.15	+0.04
0–20	5.04	5.34	+0.30	1.15	1.21	+0.06

**Table 6:** Phytomeliorative effect of safflower on agrophysical indicators of dark chestnut soils.

Soil layer (cm)	Density (g/cm^3^)			Soil structure (%)		
	Spring	Autumn	Difference	Spring	Autumn	Difference
0–10	1.31	1.30	+0.010	63.26	64.11	+0.85
10–20	1.29	1.27	+0.020	64.00	64.75	+0.75
0–20	1.30	1.29	+0.010	63.63	64.43	+0.80

In assessing the phytomeliorative role, the influence of safflower on the biological activity of dark chestnut soils is of great importance.

**Table 7:** The influence of safflower green manure on the biological activity of dark chestnut soils.

Repetitions (blank linen)	Linen weight (g) (exposure for 2 months)		The difference with the control variant due to microbiological degradation (g)	In % to control	Biological activity of the soil in comparison with the control variant (according to DG Zvyagintsev's scale)
	Initial	Final			
Control variant (blank linen)	5.3	5.3	—	100	—
1	5.3	2.15	3.15	59.43	Strong
2	5.3	2.43	2.87	54.15	Strong
3	5.2	2.33	2.87	54.15	Strong
4	5.0	2.07	2.93	55.28	Strong
Average	5.2	2.24	2.96	55.75	Strong

## Data Availability

The data used to support the findings of this study are included within the article.
